# Cancer patient satisfaction regarding the quality of information received: psychometric validity of EORTC QLQ-INFO25

**DOI:** 10.1590/0034-7167-2023-0358

**Published:** 2024-05-03

**Authors:** Michele Bezerra, Edvane Birelo Lopes De Domenico

**Affiliations:** I Universidade Federal de São Paulo. São Paulo, São Paulo, Brazil

**Keywords:** Medical Oncology, Health Education, Quality of Life, Information Management, Patient Satisfaction, Oncología Médica, Educación en Salud, Calidad de Vida, Gestión de la Información, Satisfacción del Paciente, Oncologia, Gestão da Informação, Satisfação do Paciente, Educação em Saúde, Qualidade de Vida.

## Abstract

**Objectives:**

to psychometrically validate the European Organization for Research and Treatment of Cancer Core Quality of Life Questionnaire EORTC QLQ-INFO25 instrument and identify the domains that influence patients’ perception of the information received.

**Methods:**

a cross-sectional methodology with cancer patients in a Brazilian philanthropic hospital institution. Sociodemographic and clinical instruments, EORTC QLQ-C30, EORTC QLQ-INFO25 and Supportive Care Needs Survey - Short Form 34 were used. Analysis occurred using Cronbach’s alpha coefficients, intraclass correlation, test-retest and exploratory factor analysis.

**Results:**

128 respondents participated. Cronbach’s alpha coefficient was 0.85. The test-retest obtained p-value=0.21. In the factor analysis, one item was excluded. Satisfaction with the information received was 74%, with three areas with averages below 70%. In open-ended questions, there was a greater desire for information.

**Conclusions:**

validity evidence was obtained with instrument reliability, consistency and stability. Respondents expressed satisfaction with the information received.

## RESUMO

**Objetivos:**

validar psicometricamente o instrumento da *European Organization for Research and Treatment of Cancer Core Quality of Life Questionnaire* EORTC QLQ-INFO25 e identificar os domínios que influenciam na percepção do paciente sobre as informações recebidas.

**Métodos:**

metodológico, transversal, com pacientes com câncer em instituição hospitalar filantrópica brasileira. Utilizaram-se instrumentos sociodemográficos e clínicos, EORTC QLQ-C30, EORTC QLQ-INFO25 e *Supportive Care Needs Survey - Short Form* 34. Análise ocorreu utilizando coeficientes alfa de Cronbach, correlação intraclasse, teste-reteste e análise fatorial exploratória.

**Resultados:**

participaram 128 respondentes. O coeficiente alfa de Cronbach foi de 0,85. O teste-reteste obteve p-valor=0,21. Na análise fatorial, foi excluído um item. A satisfação da informação recebida foi de 74%, com três domínios com médias inferiores a 70%. Nas questões abertas, evidenciou-se maior desejo por informações.

**Conclusões:**

evidências de validação foram obtidas com confiabilidade, consistência e estabilidade do instrumento. Os respondentes demonstraram satisfação com as informações recebidas.

## RESUMEN

**Objetivos:**

validar psicométricamente el instrumento *European Organization for Research and Treatment of Cancer Core Quality of Life Questionnaire* EORTC QLQ-INFO25 e identificar los dominios que influyen en la percepción del paciente sobre la información recibida.

**Métodos:**

metodología transversal con pacientes con cáncer en una institución hospitalaria filantrópica brasileña. Se utilizaron instrumentos sociodemográficos y clínicos, EORTC QLQ-C30, EORTC QLQ-INFO25 y *Supportive Care Needs Survey - Short Form* 34. El análisis se realizó mediante coeficientes alfa de Cronbach, correlación intraclase, prueba-reprueba y análisis factorial exploratorio.

**Resultados:**

participaron 128 encuestados. El coeficiente alfa de Cronbach fue de 0,85. La prueba-reprueba obtuvo p-valor=0,21. En el análisis factorial se excluyó un ítem. La satisfacción con la información recibida fue del 74%, con tres áreas con promedios inferiores al 70%. En las preguntas abiertas hubo mayor deseo de información.

**Conclusiones:**

se obtuvo evidencia de validación con confiabilidad, consistencia y estabilidad del instrumento. Los encuestados expresaron satisfacción con la información recibida.

## INTRODUCTION

Cancer is considered a chronic and progressive disease that causes physical, emotional and psycho-spiritual suffering^(1)^. Illness due to cancer as well as the adverse effects resulting from therapeutic modalities can trigger functional, cognitive, psychological and economic changes, affecting quality of life (QoL) and the state of organic functionality^(2)^.

There are different instruments available for assessing QoL, one of which is using instruments designed with psychometric accuracy, such as the European Organization for Research and Treatment of Cancer (EORTC) instruments. EORTC is an independent, non-profit international organization created in 1962 to coordinate research and conduct clinical trials through QoL analysis in order to improve the care of people with cancer in Europe. In 1980, EORTC created the Quality of Life Group and, in 1986, began research with the aim of assessing QoL and developing an instrument applicable to cancer patients. This led to the development of EORTC QLQ-C30^(3)^.

EORTC QLQ-C30 was developed especially for respondents with cancer and has been associated with several subscales to investigate specific situations, such as the quality of management of information received, called European Organization for Research and Treatment of Cancer Core Quality of Life Questionnaire (EORTC QLQ-INFO25). The EORTC QLQ-INFO25 module aims to assess the perception of the quality of information received by respondents with cancer, having been validated in countries such as Sweden, Spain, Germany, United Kingdom, Austria, Iran, Lebanon, Taiwan and Portugal^(4-8)^.

Assessing the quality of information received by patients is essential for their safety in managing self-care. There are positive relationships between meeting patients’ needs and their satisfaction with care as well as better symptom management, greater adherence to the therapeutic plan and better perception of QoL^(9-10)^.

Thus, the present study is justified by the need to psychometrically validate EORTC QLQ-INFO25, making it available for future national research and adding to our validity on the international stage.

## OBJECTIVES

To validate the EORTC QLQ-INFO25 instrument psychometric properties, identify the main domains that influence patients’ perception of the information received and correlate them with sociodemographic and clinical characteristics.

## METHODS

### Ethical aspects

The study was approved by the Research Ethics Committees of the *Universidade Federal de São Paulo* and the hospital institution, which hosted the study. Eligible and agreeing respondents signed the Informed Consent Form in person.

### Study design, period and place

This is a methodological, cross-sectional, quantitative study, with validity of psychometric properties of the measuring instrument^(11)^.

Data organization in this article was guided by the Standards for Quality Improvement Reporting Excellence (SQUIRE 2.0)^(12)^ checklist.

The study was carried out in the outpatient clinic and in the inpatient unit of a large, philanthropic general hospital, which has a specialized oncology center, located in the city of São Paulo, São Paulo, Brazil.

### Population: inclusion and exclusion criteria

For the present investigation, four malignant neoplasms were chosen: breast, prostate, lung and colorectal. The choice was based on the incidence of these cancers in most of the world population as well as the prospect of higher disease-free survival rates^(13-14)^.

Eligible respondents were those over 18 years of age, with one of the study’s cancer diagnoses, at any stage of the disease, including metastatic, inpatient or outpatient, undergoing treatment, with any modality.

Respondents with dementia syndromes or cognitive impairment of reading and understanding ability, certified in medical records, and who had received previous treatment for cancer in other institutions were excluded.

### Study protocol

In the present work, the translation and content validity steps were not carried out, as QLQ-INFO25 is available in the Portuguese version on the official EORTC website, for which authorization for use was requested^(15)^.

However, to improve the accuracy of this investigation, a pilot test was carried out to assess instrument comprehensibility in accordance with the EORTC Quality of Life Group Translation Procedure manual^(3)^. Five patients in outpatient care participated in the pilot test, the majority of whom were over 60 years old, female, married and Catholic, with completed higher education and economic classification B2, equivalent to the middle class. The five pilot test participants were not included to validate the questionnaire and were also excluded from the final statistical analysis. Respondents were asked about their understanding of question items and how to answer them. Everyone reported understanding the content and no difficulty in selecting the answer.

Construct validity was assessed through factor analysis, which aims to summarize the information contained in several variables with the aim of minimizing information loss^(16)^. For convergent validity, which aims to compare the instruments to confirm whether they both measure the same objectives, the Supportive Care Needs Survey - Short Form 34 (SCNS-SF34), Brazilian version, was chosen^(17)^.

Reliability was assessed through internal consistency and stability. Internal consistency was verified using Cronbach’s alpha coefficient and stability using the Intraclass Correlation Coefficient (ICC)^(18)^. In the test-retest, the questionnaire was applied on two separate occasions, however, to half of respondents, due to the COVID-19 pandemic triggered during the data collection period.

Sample calculation was carried out based on the recommendations made by Hair^(16)^ to include 5 to 10 respondents per item of the questionnaire under study for a 5% level of significance, estimating at least 125 participants.

### Instruments

a) Questionnaire with sociodemographic and clinical characteristics.

b) QLQ-C30: the instrument formulated by EORTC assesses cancer survivors’ QoL. Contains 30 items, divided into five functional scales, three symptom scales, six single-item questions and one item on health status/overall QoL^(3)^.

c) QLQ-INFO25: is a self-report instrument that aims to measure patients’ perception of the information received. The instrument has 25 questions, which are measured using a 4-point Likert scale (none, little, reasonable, quite a lot), except for questions 50, 51, 53 and 54, which are dichotomous “yes” and “no” questions. Scores obtained on a Likert scale are linearly transformed into 0-100. Higher scores mean a higher level of information received^(15,19)^.

QLQ-INFO25 presents the respective domains: InfoDIS (Information about the disease); InfoMedt (Information about medical tests); Infotreadt (Information about treatments); InfoThse (Information about other services); InfoDifp (Information about different places of care); InfoHelp (Information about things you can do to help yourself); InfoWrin (Written information); InfoCD (Information on CD/tape/video); Satinfo (Satisfaction with the information received); Recmore (Desire to receive more information); Recless (Desire to receive less information); Overhelp (Usefulness of information)^(15)^.

d) SCNS-SF34: the instrument assesses the different areas of needs for cancer patients, consisting of 34 items. In the validated Brazilian version, there are seven domains (*Físico e vida diária, Psicológico, Controle e visão positiva, Sexualidade, Cuidado e suporte, Sistema de saúde e Informação*) and 4 individual items^(17)^.

### Analysis of results, and statistics

Sociodemographic data analysis was descriptive, in absolute numbers and percentages, using the software Statistical Package for the Social Sciences (SPSS) version 20, Minitab 16 and Excel Office 2010. Construct analysis was carried out using the exploratory factor analysis (EFA) factor analysis approach. It was not possible to perform confirmatory factor analysis (CFA), because there were no variations in responses. Previously, the Kaiser-Meyer-Olkin (KMO) and Bartlett tests were used.

Reliability analysis was performed using Cronbach’s alpha coefficient. Stability was assessed using ICC and Spearman-Brown correlation. To compare the results of each protocol score between test-retest, the non-parametric Wilcoxon test was used.

EORTC QLQ-C30 questionnaires and EORTC-INFO25 module were calculated by linear transformation with scores from 0 to 100, as described in the EORTC manual.

The two open-ended questions contained in EORT-INFO25 were analyzed using content analysis proposed by Laurence Bardin. Bardin’s method was used in its three parts: pre-analysis, material exploration and treatment of results (inferences and interpretations)^(20)^. Qualitative data interpretation was based on the concepts of health education in chronic illness and the process of becoming ill due to cancer.

The first question follows the item “Would you like to receive more information?”; the second question relates to the item “Would you like to receive less information?”; and in both, it is written as follows: “If yes, please specify on which subjects”. As there is no express guidance in the manual for organizing this data, we chose to use the ATLAS.ti^®^ software (Scientific Software Development, Berlin, Germany)^(21-22)^, ATLAS.ti^®^ 8.0 student license (version 8.4.24.0).

## RESULTS

A total of 128 respondents participated, who were in inpatient (23.4%) and outpatient (76.5%) units. In Table 1, the sociodemographic characteristics are described.

**Table 1:** Respondent data regarding sociodemographic characteristics, São Paulo, São Paulo, Brazil

Variable	Category	Frequency	Percentage
Sex	F	78	60.90%
	M	50	39.10%
Age	< 60	53	41.00%
	≥60	75	59.00%
Education	Elementary school	1	0.80%
	High school	19	11.90%
	Higher education	108	84.40%
	Married	104	81.30%
Marital status	Divorced/single/widowed	24	18.70%
	Catholic	93	72.70%
Religion	Others	35	27.30%
Profession	Self-employed professional	78	61.00%
	Health professional	17	13.30%
	Housewife	13	10.10%
	Others	20	15.60%
Socioeconomic classification	A	14	10.90%
	B1/B2	86	67.20%
	C1/C2	28	21.90%
Total		128	100%

There was a predominance of females (60.9%), married people (81.3%) and Catholics (72.7%). Regarding education, 84.4% had completed higher education, and the prevalence of socioeconomic classification found was medium income B1/B2 (67.20%).

In Table 2, there are clinical data from the validity series.

**Table 2:** Respondent data regarding clinical characteristics, São Paulo, São Paulo, Brazil

Variable	Category	Frequency	Percentage
Smoking	Former smoker	35	27.30%
	Smoker	4	3.10%
	Non-smoker	89	69.50%
	Diabetes Mellitus	26	20.30%
	Hypertension	24	18.8%
Comorbidities	Others^*^	8	6.3%
	No	70	54.60%
Oncological disease	Colorectal cancer	39	30.45%
	Breast cancer	45	35.15%
	Prostate cancer	14	11%
	Lung cancer	30	23.40%
Time since diagnosis	Less than 6 months	37	28.90%
	7-12 months	47	36.80%
	Over 13 months	44	34.30%
Antineoplastic treatment	Intravenous (IV) antineoplastic chemotherapy	110	86.00%
	IV and oral antineoplastic chemotherapy	9	7.00%
	Oral endocrine therapy	9	7.00%
Radiotherapy	No	77	60.20%
	Yes	51	39.80%
Surgery	No	28	21.90%
	Yes	100	78.10%
Total		128	100%

* *Dyslipidemia, chronic obstructive pulmonary disease, hypothyroidism, systemic lupus erythematosus, osteoporosis, Parkinson’s, bipolar disorder, panic syndrome, acute myocardial infarction, heart disease.*

Regarding cancer diagnoses, there was a higher prevalence of breast cancer (35.15%). Regarding the treatment performed, intravenous antineoplastic chemotherapy was predominant (86%), and, in relation to comorbidities, the majority (54.6%) did not present them.

The result of the KMO test was 0.782, enabling the data to carry out factor analysis. The Bartlett test was significant (<0.001), i.e., showing that there was a correlation between the data. The Varimax orthogonal rotation method with “Kaiser” normalization was used.

Factor analysis was performed using the principal components method, obtaining factors with eigenvalues greater than 1. For each factor, the variability explained by that factor was found, in addition to accumulated variability. However, question Q51 “Did you receive information on CD or tape/video?” was disregarded for this test, as it showed no variability, i.e., the answers were 4: not executable.

It was found that the 25 questions generated 7 factors (groups of questions), in which the total variability explained by these was 77.3% (of the total 100%), which is considered a significance value. To conclude EFA, in Table 3, the factor loadings for each of the questions in each of the factors are presented.

**Table 3:** Factor loading of questions in each factor^*^, São Paulo, São Paulo, Brazil

	Factor 1	Factor 2	Factor 3	Factor 4	Factor 5	Factor 6	Factor 7
Q48	0.932						
Q49	0.932						
Q47	0.868						
Q46	0.800						
Q45	0.573						
Q44	0.547						
Q35		0.965					
Q36		0.965					
Q37		0.948					
Q39			0.962				
Q31			0.962				
Q38			0.891				
Q55			0.546				
Q42				0.814			
Q43				0.724			
Q41				0.675			
Q40				0.594			
Q32				-0.368			
Q34					0.752		
Q33					0.724		
Q54						0.812	
Q52						0.623	
Q53						0.591	
Q50							0.833

* *Extraction method: principal component analysis/rotation method: Varimax with Kaiser normalization. Rotation converged in 9 interactions.*

According to the data above, it was observed that the results converged with only 11 interactions, and the result of the main factor, i.e., factor 1, which alone held 26.4% of data variability, was relevant to the questions 48, 49, 47, 46, 45 and 44. Among these six questions, the most important was question 48 (“Other places for medical care hospitals/outpatient clinics/at home”), which had a factor loading of 0.932. These results indicate that the greater the load, the greater the representativeness of the question for the factor.

It was found that the EORTC QLQ-INFO25 protocol consistency presented a Cronbach’s alpha of 0.85. Thus, it is stated that the protocol is consistent. To analyze QLQ INFO25 reliability, test-retest was used on 50% of the sample. For this, the Wilcoxon test was used, with no statistical difference between the test-retest in all scores, including the overall score, in which the mean was 74.86 in the test against 75.35 in the retest (p-value = 0.213), concluding that the protocol is reproductive.

QLQ INFO25 stability was also verified by convergent validity with SCNS-SF34, through ICC, with values of 0.571, limit of 0.470-0.663 for QLQ Info-25 and 0.843, limit of 0.786-0.875 for SCNS-SF34 and Spearman-Brown correlation (r = 0.762 and 0.872; p = 0.865 and 0.932, for QLQ INFO-25 and SCNS-SF34). The values found were statistically significant, indicating agreement.

**Figure 1:**
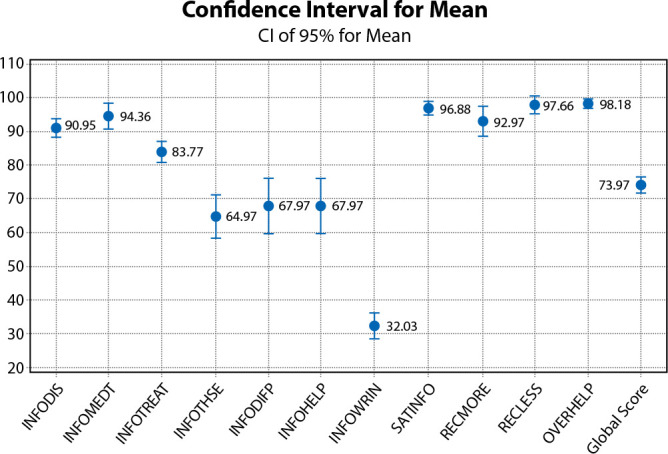
Confidence interval for the QLQ INFO-25 mean, São Paulo, São Paulo, Brazil

Figure 1 presents the results of QLQ Info-25 analysis regarding the domains with mean, showing an overall score of 73.97%, i.e., respondents’ satisfaction in relation to the information received. However, there were domains whose approval means were lower than 70%, such as InfoThse (Information on other services), InfoDifp (Information about different places of care), InfoHelp (Information about things you can do to help yourself) and InfoWrin (Written information), lower than 50%.

Finally, the overall QLQ INFO-25 score was evaluated with demographic and clinical variables. The Mann-Whitney test (when the variable has only two response levels) and the Kruskal-Wallis test (when the variable has three or more response levels) were used. The results ranged from 0.096 to 0.889, leading to the conclusion that there is no statistical significance in any of the demographic covariates, i.e., none of these demographic variables had an effect on the overall result of QLQ INFO-25.

Subsequently, respondents’ satisfaction with the information received was assessed, correlating it with sociodemographic and clinical variables.

Assessing the relationship between satisfaction and age, sex, education, smoking, comorbidities, economic classification, oncological disease, radiotherapy treatment, surgery and antineoplastic chemotherapy treatment, there was no significant difference.

In the analysis regarding religion, there was a significant difference in relation to the Recmore domain p 0.035 (“Desire to receive more information”), in which respondents who declared themselves without religion were less satisfied, when compared to Catholics and other religions.

In the correlation with profession and economic classification, in the profession item, there was a significant difference in the Overhelp domain (“Usefulness of information”). Regarding this domain, people who referred to themselves as “at home” expressed that the information received during treatment was not sufficiently useful, compared to self-employed professionals and other professions.

A significant difference was evident in the domains InfoThse (“Information about other services”) (p 0.034) and Satinfo (“Satisfaction with the information received”) (p 0.021), in which respondents with a diagnosis time between 7-12 months achieved a high mean, being more satisfied than those with less than 7 months and more than 12 months.

In open-ended questions, respondents were able to express their desire to receive more or less information, making it possible to identify the aspects that required greater concern during the treatment journey. The documents with participants’ testimonies, in full, were added to the ATLAS.ti^®^ 8.0 software and generated the data for Chart 1. In the column called ID, the first number corresponds to the respondent’s document and the sequence number corresponds to the number of codes that the quote linked to. In the second column, there are the full citations and, in the third column, the inferential interpretative analyzes.

**Chart 1:** Patients’ opinions on the topics on which they would like to receive more or less information during antineoplastic treatment

ID	Citation content	Inferential analysis
2:1	“I would like to know more details about: whether you can return; what is the probability; what other types exist in the same location.”	Desire to know more: occurrence of recurrence/other types of cancer in the same location
3:1	“Inform more about radiotherapy, survival and care at home.”	Desire to know more: radiation therapy/survival time/ cancer survivorship care
4:1	“Knowing if treatment was successful, how long you have to live.”	Desire to know more: treatment results/survival time
5:1	“Knowing if treatment worked.”	Desire to know more: treatment results/survival time
6:1	“Reconciliation between commonly used medications and new treatments.”	Desire to know more: medication conciliation (previous drugs and new drugs)
7:1	“To find out more about the interaction with the medications I started taking along with chemotherapy and when it ends, what will it be like?”	Desire to know more: drug interaction/ cancer survivorship care
8:1	“Knowing after treatment what will happen.”	Desire to know more: cancer survivorship care
9:1	“I’m feeling a little better, but I still want to know more and more, to feel safe.”	Desire to know more: information capable of generating security
10:1	“I still have a little doubt about home care, what I can do.”	Desire to know more: home care/security/cancer survivorship care
11:1	“About marital relationship issues /About whether I can use the same things with my son and family.”	Desire to know more: sexuality/relationships/security in family life
12:1	“I feel unsure of what to do from now on with the symptoms.”	Desire to know more: treatment results/ cancer survivorship care
13:1	“Explain more about the transition from chemotherapy to immunotherapy /Sometimes there’s a lot of noise and you have to repeat it more often/Explain why I can’t travel /Too many things at the same time.”	Desire to know more: stages and types of treatment/drugs/safety/restrictions. Information in suitable and planned environments/gradual information
14:1	“Whether I’ll ever have it again.”	Desire to know more: recurrence/fear/expectation
15:1	“About home care, social life.”	Desire to know more: home care/safety/social interactions
16:1	“I don’t want to know too much.”	Desire to know less: denial/ineffective coping
17:1	“I don’t want to know too much.”	Desire to know less: denial/ineffective coping

## DISCUSSION

The methodological study that gave rise to EORTC-INFO25 generated translated versions for 25 languages that favor the comparative analysis of studies around the world, such as English, Portuguese from Brazil and Portugal, Arabic, Spanish, German, Italian, Mandarin and French^(3)^. The steps carried out in the present investigation enabled psychometric validity for use in the national territory.

The KMO test enabled factor analysis. In the factorial analysis of principal components, only question Q51 (“Did you receive information on CD or tape/video?”) was disregarded, as it did not present variability, and in EFA, the results converged.

When comparing the Cronbach’s alpha of this study with the Spanish, Arabic (Lebanese), Iranian versions and the version that evaluated 7 European countries and Taiwan, a similar alpha of 0.85, 0.90, 0.920, 0.91 and ≥ 0.7, respectively. Correlating the alpha of the factors, very close values were also found. Thus, it can be considered that the Brazilian version of QLQ INFO25 demonstrated high reliability^(4,6-8)^.

The INFO25 test-retest showed no significant difference in relation to the two moments of application, indicating instrument stability^(18)^.

The convergent validity presented a satisfactory result, since EORTC-INFO25 correlated with SCNS-SF34 through ICC and Spearman-Brown correlation.

In the present investigation, the predominance of respondents was female, with more cases of breast and colorectal cancer, which is consistent with incidence statistics by sex in Brazil, with breast and colorectal cancer occupying, respectively, the first and second position of incidence in women^(13)^.

Regarding the professional profile, a greater number of self-employed professionals was evident, which corresponds, in a certain way, to the predominance of higher strata classes (A and B combined) and level of education in higher education. This educational socioeconomic profile is consistent with the supplementary healthcare population in Brazil, according to data from the Brazilian National Supplementary Health Agency (ANS - *Agência Nacional de Saúde Suplementar*), which analyzed, in January 2021, an increase in the number of healthcare plan beneficiaries, highlighting the importance of the sector and Brazilians’ interest in access to supplementary healthcare^(23)^.

In the responses obtained with the application of EORTC-INFO25, there was no difference in patients’ perception regarding the information received between sexes, unlike the Spanish study, which showed that women would like to receive more information^(7)^. When comparing the scale domains with age, it was observed that there was no difference between those over or under 60 years of age as well as there was no significant difference in male and female patient perceptions. Study carried out to validate EORTC-INFO25 in patients with different types of cancer and stages of the disease, which evaluated seven European countries and Taiwan, showed that patients under the age of 50 had a greater desire for information than older patients, when assessing information about treatments, other services and written information^(8)^.

In relation to religion, there was a statistical difference in the Recmore domain p 0.035 (“Desire to receive more information”), in which people who declared themselves without religion were less satisfied when compared to Catholics and other religions. The religiosity and spirituality of cancer patients are positive predictors of coping, resilience and hope^(24)^.

According to the professional profile data, there was a significant difference in the Overhelp domain (“Usefulness of information”) for people who referred to themselves as “at home”, compared to self-employed professionals and those from other professions. People who declared themselves “at home” expressed that the information received during treatment was not useful enough. One of the possibilities to explain this finding is in the social representation of these women of already having full control over disease care, since they are exclusively responsible for family care^(25)^.

For the time of diagnosis, it was found that patients undergoing treatment for 7-12 months were more satisfied, compared to those with a period of less than 7 months and more than 12 months. Studies on the impact of cancer on patients’ mental health and well-being indicate that depression and anxiety can hinder treatment and recovery as well as QoL and survival, and must be diagnosed early so that patients can face each phase of the cancer continuum with the necessary skills^(26-27)^.

It was observed that the greatest demands of patients’ needs in relation to all sociodemographic and clinical variables studied were: Infothse (“Information about other services”); InfoDifp (“Information about different places of care”); InfoHelp (“Information about things you can do to help yourself”); and InfoWrin (“Written Information”). In the Lebanese study, patients reported receiving less information about rehabilitation services (86%), out-of-hospital services (83.6%), and sexual activity (83.6%), but overall, patients assumed were satisfied with the information provided (86%), and 89.5% stated that the information was useful^(4)^.

Question Q51 “Did you receive information on CD or tape/video?” did not receive any response, indicating that all patients mentioned that this CD or tape/video resource was not used as an educational method. Certainly, this item presents a certain obsolescence nowadays, due to the wide use of modern technological resources for health education, such as social media or digital and collaborative education platforms via the web^(28-29)^.

Regarding open-ended questions, few patients responded and the desire for more information was broad, including survival, treatment, care and safety. Health education for people with chronic illness provides for the development of self-management skills, favoring shared decision-making with the healthcare team, symptom management and necessary behavioral changes. The breadth of content highlighted reiterates the need to plan educational action for the multidimensional care of people with cancer, considering the most delicate aspects, such as prognosis, fear of disease recurrence and treatment sequels^(30-31)^.

### Study limitations

The present study has limitations, such as difficulty in expanding the sample composition due to periods of discontinuity in data collection due to the COVID-19 pandemic and characterization of an educational socioeconomic profile that does not represent the national reality. Therefore, it is advisable to reassess instrument internal consistency in future investigations whose participants present a social vulnerability profile.

### Contributions to nursing

The EORTC-INFO25 is easy to apply and understand, and can be used to monitor and evaluate the quality of health educational action, from the perspective of patients who are on their cancer treatment journey. The results generated by its application allow evaluating the information provided to cancer patients, considering several domains that highlight the processes that require improvement for the qualification of educational action in health, led, in most multidisciplinary teams, by nurses.

## CONCLUSIONS

In the analysis of the psychometric properties for instrument validity, it was found that the protocol is consistent and has high reliability. EFA confirmed the correlation between the data. The test-retest, applied in two moments, did not show a significant difference, indicating instrument stability. In convergent assessment, EORTC-INFO25 correlated with SCNS-SF34.

The results generated by its application showed that the main domains that influenced patients’ perception of the information received were: Infothse (“Information about other services”); InfoDifp (“Information about places of care”); InfoHelp (“Information about things you can do to help yourself”); and InfoWrin (“Written Information”). In open-ended questions, there was a greater desire for information about the risk of failure (progression) and/or recurrence, survival and care at home.
